# Xyloccensin L

**DOI:** 10.1107/S1600536810028527

**Published:** 2010-07-24

**Authors:** Gang Feng, Jing Zhang, Jun Li, Tirumani Satyanandamurty, Jun Wu

**Affiliations:** a Environment and Plant Protection Institute, Chinese Academy of Tropical Agricultural Science, Danzhou 571737, People’s Republic of China; b Key Laboratory of Marine Bio-resources Sustainable Utilization, South China Sea Institute of Oceanology, Chinese Academy of Sciences, 164 West Xingang Road, Guangzhou 510301, People’s Republic of China; c Government Degree College at Amadala Valasa, Srikakulam District, Andhra Pradesh, 532185, India

## Abstract

The title compound, C_32_H_40_O_10_, also known as xyloccensin L [systematic name: (1*R*,4a*R*,4b*S*,5a*R*,6a*R*,9*R*,10*S*,10a*S*,10b*R*,2a*R*,13*R*)-1-(furan-3-yl)-6a-hy­droxy-10-(2-meth­oxy-2-oxoeth­yl)-9,10a,12a-trimethyl-3-oxododeca­hydro-1*H*,3*H*,5a*H*-6,9-methano­isochromeno[6,5-*f*]oxireno[*g*]chromen-13-yl (2*E*)-2-methyl­but-2-enoate], is a limonoid with a C1—C29 oxygen bridge: this is the first report of the X-ray crystal structure of such a limonoid. Two fused pyran rings and two cyclo­hexane rings adopt boat conformations, while another cyclo­hexane ring and the *d*-lactone ring are in half-chair conformations. The mol­ecular structure is stabilized by intra­molecular O—H⋯O hydrogen bonding.

## Related literature

The title compound was isolated from seeds of an Indian mangrove, *Xylocarpus moluccensis*, collected in the mangrove wetlands of the Godavari estuary, Andhra Pradesh. For previous investigations of the seeds of *Xylocarpus granatum* and *X. moluccensis*, see: Kubo *et al.* (1976 ▶); Ng *et al.* (1979 ▶); Alvi *et al.* (1991 ▶); Kokpol *et al.* (1996 ▶); Mulholland *et al.* (2000 ▶). For our group’s work in this field, see: Wu *et al.* (2004*a* ▶,*b* ▶, 2005 ▶, 2008*a* ▶,*b* ▶).
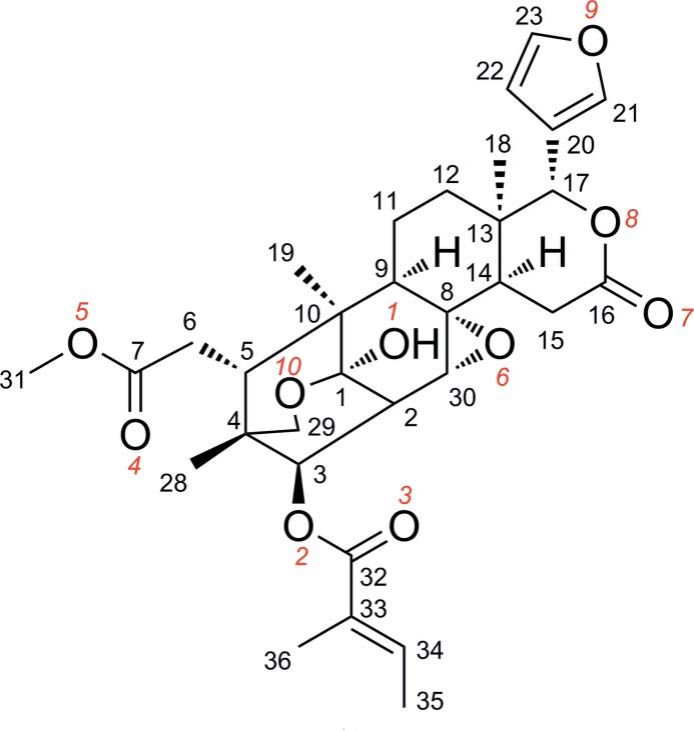


## Experimental

### 

#### Crystal data

C_32_H_40_O_10_
*M* *_r_* = 584.64
Orthorhombic, 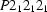

*a* = 8.3859 (4) Å
*b* = 11.0454 (5) Å
*c* = 31.0799 (13) Å
*V* = 2878.8 (2) Å^3^
*Z* = 4
Mo *K*α radiation
μ = 0.10 mm^−1^
*T* = 110 K
0.46 × 0.44 × 0.25 mm

#### Data collection

Bruker SMART 1000 CCD diffractometer
Absorption correction: multi-scan (*SADABS*; Bruker, 2004 ▶) *T*_min_ = 0.956, *T*_max_ = 0.976
14701 measured reflections
3569 independent reflections
3202 reflections with *I* > 2σ(*I*)
*R*_int_ = 0.026

#### Refinement

*R*[*F* ^2^ > 2σ(*F* ^2^)] = 0.036
*wR*(*F* ^2^) = 0.096
*S* = 1.03
3569 reflections
386 parameters
H-atom parameters constrained
Δρ_max_ = 0.59 e Å^−3^
Δρ_min_ = −0.20 e Å^−3^

### 

Data collection: *SMART* (Bruker, 2004 ▶); cell refinement: *SAINT-Plus* (Bruker, 2004 ▶); data reduction: *SAINT-Plus*; program(s) used to solve structure: *SHELXS97* (Sheldrick, 2008 ▶); program(s) used to refine structure: *SHELXL97* (Sheldrick, 2008 ▶); molecular graphics: *SHELXTL* (Sheldrick, 2008 ▶); software used to prepare material for publication: *SHELXL97* .

## Supplementary Material

Crystal structure: contains datablocks I, global. DOI: 10.1107/S1600536810028527/ez2224sup1.cif

Structure factors: contains datablocks I. DOI: 10.1107/S1600536810028527/ez2224Isup2.hkl

Additional supplementary materials:  crystallographic information; 3D view; checkCIF report

## Figures and Tables

**Table 1:** Hydrogen-bond geometry (Å, °)

*D*—H⋯*A*	*D*—H	H⋯*A*	*D*⋯*A*	*D*—H⋯*A*
O1—H1⋯O6	0.84	2.08	2.774 (2)	139

**Fig. 1.**
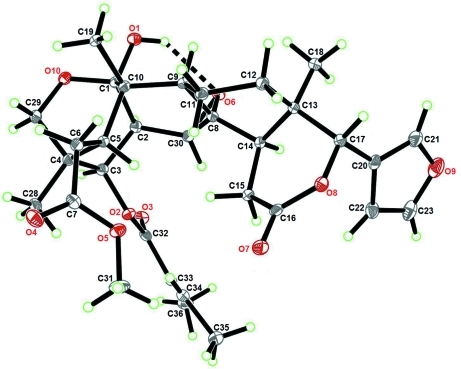
The molecular structure of the title molecule, with the atom-numbering scheme. Displacement ellipsoids are drawn at the 30% probability level. The intramolecular hydrogen bond is shown as a dashed line.

**Fig. 2.**
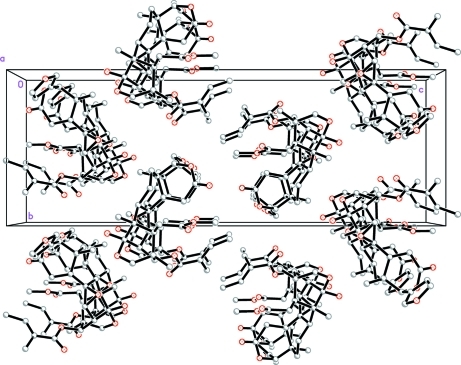
The crystal packing of (I), viewed down the a axis.

## supplementary crystallographic information

### Comment

Two meliaceous mangroves, *Xylocarpus granatum* and *X*. *moluccensis*, are known for producing antifeedant limonoids, especially mexicanolides and phragmalins. Previous investigations on seeds of these two species, yielded an andirobin, an obacunol, two phragmalins, three gedunins, and 14 mexicanolides, including xyloccensins A-K (Kubo *et al.*, 1976; Ng *et al.*, 1979; Alvi *et al.*, 1991; Kokpol *et al.*, 1996; Mulholland *et al.*, 2000). Previously, we reported the isolation and identification of eight unique 8,9,30-phragmalin ortho esters and 13 limonoids from the bark and seeds of a Chinese mangrove, *X. granatum* (Wu *et al.*, 2004*a*, 2005, 2008*a*). To date, altogether 42 mexicanolides and 23 phragmalins have been isolated from the wood, seeds, and fruits of *X. granatum* and *X. moluccensis* (Wu *et al.*, 2008*b*).

The title compound (I), also known as xyloccensin L (Wu *et al.*, 2004*b*), was previously isolated from seeds of a Chinese mangrove, *X. granatum*, which was collected from the Hainan island. As part of our research on bioactive compounds from mangrove plants of the Xylocarpus genus, we obtained the title compound again from seeds of an Indian mangrove, *X. moluccensis*, collected in the mangrove wetlands of Godavari estuary, Andhra Pradesh. The X-ray crystal structure analysis of (I) was undertaken in order to establish its relative stereochemistry and confirm our previously reported stereostructure (Wu *et al.*, 2004*b*). Two fused pyran rings, C1/C2/C3/C4/C29/O10 and C1/C10/C5/C4/C29/O10, and two cyclohexane rings, C1/C2/C3/C4/C5/C10 and C8/C9/C11/C12/C13/C14, adopt boat conformations. However, the cyclohexane ring C1/C2/C30/C8/C9/C10 and the d-lactone ring C13/C14/C15/C16/O8/C17 are in half-chair conformations. In the crystal structure, molecules are stabilized by O—H···O intramolecular hydrogen bonds between O1/O6 (Figure 1). The whole shape of the molecule is like a cage. O1 and O10 are at the bottom of the cage, whereas the furan ring and the 3-tigloyl group are on the top of the cage. Molecules are arranged like cages in an alternating bottom-to-bottom and top-to-top fashion along the *b* axis (Fig. 2).

### Experimental

Dried seeds (8.7 kg) of *X. moluccensis* were extracted three times with 95% EtOH at room temperature. The extract was concentrated under reduced pressure, followed by suspension in H_2_O and extraction with EtOAc. The resulting EtOAc extract (198.0 g) was chromatographed on silica gel eluted using a CHCl_3_-MeOH system (100:0 - 5:1) to yield 127 fractions. Fractions 47 to 62 (66.2 g) were combined and further separated using RP C_18_ CC (MeCN-H_2_O, 50:50 - 100:0) to afford 132 subfractions. Then subfractions 13 to 15 were combined and subjected to preparative HPLC [YMC-Pack ODS-5-A, 250×20 mm i.d. (MeOH-H_2_O, 57 : 43), and 250×10 mm i.d. (MeCN-H_2_O 43 : 57)] to yield the title compound I (15.0 mg).

### Refinement

All non-hydrogen atoms were refined anisotropically. All the H atoms were placed in geometrically idealized positions (C—H = 0.98 Å, with *U*_iso_(H) = 1.5*U*_eq_(C) for methyl groups; C—H = 0.99 Å, with *U*_iso_(H) = 1.2*U*_eq_(C) for methylene groups; C—H = 0.95 Å, with *U*_iso_(H) = 1.2*U*_eq_(C) for aromatic rings; C-H = 0.95 Å, with Uiso(H) = 1.2Ueq for alkyne group, O—H = 0.84 Å, with *U*_iso_(H) = 1.5*U*_eq_(O) for hydroxyl group) and constrained to ride on their parent atoms. In the absence of significant anomalous scattering effects 2707 Friedel pairs have been merged.

### Crystal data

**Table:**

C_32_H_40_O_10_	*F*(000) = 1248
*M**_r_* = 584.64	*D*_x_ = 1.349 Mg m^−^^3^
Orthorhombic, *P*2_1_2_1_2_1_	Mo *K*α radiation, λ = 0.71073 Å
Hall symbol: P 2ac 2ab	Cell parameters from 7947 reflections
*a* = 8.3859 (4) Å	θ = 2.3–27.0°
*b* = 11.0454 (5) Å	µ = 0.10 mm^−^^1^
*c* = 31.0799 (13) Å	*T* = 110 K
*V* = 2878.8 (2) Å^3^	Block, colorless
*Z* = 4	0.46 × 0.44 × 0.25 mm

### Data collection

**Table:**

Bruker SMART 1000 CCD diffractometer	3569 independent reflections
Radiation source: fine-focus sealed tube	3202 reflections with *I* > 2σ(*I*)
graphite	*R*_int_ = 0.026
ω scans	θ_max_ = 27.0°, θ_min_ = 2.0°
Absorption correction: multi-scan (*SADABS*; Bruker, 2004)	*h* = −10→8
*T*_min_ = 0.956, *T*_max_ = 0.976	*k* = −11→14
14701 measured reflections	*l* = −39→29

### Refinement

**Table:**

Refinement on *F*^2^	Primary atom site location: structure-invariant direct methods
Least-squares matrix: full	Secondary atom site location: difference Fourier map
*R*[*F*^2^ > 2σ(*F*^2^)] = 0.036	Hydrogen site location: inferred from neighbouring sites
*wR*(*F*^2^) = 0.096	H-atom parameters constrained
*S* = 1.03	*w* = 1/[σ^2^(*F*_o_^2^) + (0.0574*P*)^2^ + 0.880*P*] where *P* = (*F*_o_^2^ + 2*F*_c_^2^)/3
3569 reflections	(Δ/σ)_max_ = 0.002
386 parameters	Δρ_max_ = 0.59 e Å^−^^3^
0 restraints	Δρ_min_ = −0.19 e Å^−^^3^

### Special details

**Table:**

Geometry. All esds (except the esd in the dihedral angle between two l.s. planes) are estimated using the full covariance matrix. The cell esds are taken into account individually in the estimation of esds in distances, angles and torsion angles; correlations between esds in cell parameters are only used when they are defined by crystal symmetry. An approximate (isotropic) treatment of cell esds is used for estimating esds involving l.s. planes.
Refinement. Refinement of F^2^ against ALL reflections. The weighted R-factor wR and goodness of fit S are based on F^2^, conventional R-factors R are based on F, with F set to zero for negative F^2^. The threshold expression of F^2^ > 2sigma(F^2^) is used only for calculating R-factors(gt) etc. and is not relevant to the choice of reflections for refinement. R-factors based on F^2^ are statistically about twice as large as those based on F, and R- factors based on ALL data will be even larger.

### Fractional atomic coordinates and isotropic or equivalent isotropic displacement parameters (Å^2^)

**Table:**

	*x*	*y*	*z*	*U*_iso_*/*U*_eq_	
C1	0.5981 (3)	0.5741 (2)	0.22231 (7)	0.0195 (4)	
C2	0.7232 (3)	0.6275 (2)	0.19155 (7)	0.0182 (4)	
H2	0.7917	0.6848	0.2082	0.022*	
C3	0.6325 (3)	0.7006 (2)	0.15695 (7)	0.0189 (5)	
H3	0.6422	0.7889	0.1635	0.023*	
C4	0.4563 (3)	0.6652 (2)	0.15701 (7)	0.0179 (4)	
C5	0.4478 (3)	0.5242 (2)	0.15534 (7)	0.0164 (4)	
H5	0.5271	0.4973	0.1333	0.020*	
C6	0.2853 (3)	0.4755 (2)	0.14117 (7)	0.0191 (4)	
H6A	0.2777	0.3897	0.1501	0.023*	
H6B	0.2015	0.5207	0.1568	0.023*	
C7	0.2499 (3)	0.4829 (2)	0.09360 (7)	0.0200 (5)	
C8	0.7803 (3)	0.4010 (2)	0.17338 (7)	0.0174 (4)	
C9	0.6201 (3)	0.36277 (19)	0.19238 (7)	0.0172 (4)	
H9	0.6436	0.3283	0.2214	0.021*	
C10	0.5048 (3)	0.4712 (2)	0.19984 (7)	0.0169 (4)	
C11	0.5487 (3)	0.2586 (2)	0.16552 (7)	0.0184 (4)	
H11A	0.4458	0.2333	0.1783	0.022*	
H11B	0.5280	0.2874	0.1359	0.022*	
C12	0.6618 (3)	0.1505 (2)	0.16406 (8)	0.0212 (5)	
H12A	0.6235	0.0933	0.1418	0.025*	
H12B	0.6574	0.1084	0.1921	0.025*	
C13	0.8391 (3)	0.1833 (2)	0.15411 (7)	0.0187 (5)	
C14	0.8571 (3)	0.31658 (19)	0.14091 (7)	0.0170 (4)	
H14	0.9738	0.3352	0.1406	0.020*	
C15	0.7927 (3)	0.3404 (2)	0.09497 (7)	0.0187 (4)	
H15A	0.6988	0.3943	0.0975	0.022*	
H15B	0.8755	0.3853	0.0788	0.022*	
C16	0.7446 (3)	0.2319 (2)	0.06835 (7)	0.0196 (5)	
C17	0.8969 (3)	0.1031 (2)	0.11665 (7)	0.0198 (5)	
H17	0.8806	0.0173	0.1259	0.024*	
C18	0.9417 (3)	0.1573 (2)	0.19414 (7)	0.0252 (5)	
H18A	1.0508	0.1857	0.1891	0.038*	
H18B	0.9429	0.0700	0.1998	0.038*	
H18C	0.8966	0.1997	0.2190	0.038*	
C19	0.3680 (3)	0.4315 (2)	0.22968 (7)	0.0200 (5)	
H19A	0.4090	0.4204	0.2589	0.030*	
H19B	0.3230	0.3551	0.2193	0.030*	
H19C	0.2848	0.4939	0.2298	0.030*	
C20	1.0686 (3)	0.1145 (2)	0.10408 (8)	0.0229 (5)	
C21	1.1915 (3)	0.0517 (3)	0.12110 (9)	0.0388 (7)	
H21	1.1806	−0.0064	0.1435	0.047*	
C22	1.1390 (3)	0.1892 (2)	0.07166 (9)	0.0291 (6)	
H22	1.0848	0.2445	0.0534	0.035*	
C23	1.2970 (3)	0.1660 (2)	0.07200 (10)	0.0352 (6)	
H23	1.3731	0.2034	0.0536	0.042*	
C28	0.3676 (3)	0.7303 (2)	0.12087 (7)	0.0227 (5)	
H28A	0.2525	0.7192	0.1246	0.034*	
H28B	0.4003	0.6966	0.0931	0.034*	
H28C	0.3931	0.8168	0.1217	0.034*	
C29	0.3932 (3)	0.7090 (2)	0.20030 (7)	0.0216 (5)	
H29A	0.3890	0.7986	0.2004	0.026*	
H29B	0.2834	0.6783	0.2046	0.026*	
C30	0.8289 (3)	0.5291 (2)	0.17323 (7)	0.0186 (4)	
H30	0.9032	0.5542	0.1497	0.022*	
C31	0.3549 (4)	0.4634 (3)	0.02342 (7)	0.0309 (6)	
H31A	0.2752	0.4020	0.0160	0.046*	
H31B	0.4555	0.4450	0.0088	0.046*	
H31C	0.3168	0.5433	0.0143	0.046*	
C32	0.8270 (3)	0.7374 (2)	0.10287 (7)	0.0201 (5)	
C33	0.8800 (3)	0.7130 (2)	0.05808 (7)	0.0217 (5)	
C34	0.8116 (3)	0.6249 (2)	0.03490 (7)	0.0239 (5)	
H34	0.7187	0.5880	0.0464	0.029*	
C35	0.8694 (4)	0.5802 (3)	−0.00750 (8)	0.0334 (6)	
H35A	0.9248	0.6458	−0.0226	0.050*	
H35B	0.7784	0.5530	−0.0248	0.050*	
H35C	0.9430	0.5124	−0.0030	0.050*	
C36	1.0232 (3)	0.7845 (3)	0.04405 (8)	0.0308 (6)	
H36A	1.1197	0.7488	0.0565	0.046*	
H36B	1.0125	0.8684	0.0539	0.046*	
H36C	1.0309	0.7831	0.0126	0.046*	
O1	0.6661 (2)	0.53802 (16)	0.26154 (5)	0.0239 (4)	
H1	0.7477	0.4960	0.2567	0.036*	
O2	0.69261 (19)	0.67773 (14)	0.11384 (5)	0.0196 (3)	
O3	0.8976 (2)	0.80205 (15)	0.12781 (5)	0.0266 (4)	
O4	0.1193 (2)	0.50008 (15)	0.07891 (6)	0.0272 (4)	
O5	0.3803 (2)	0.46342 (16)	0.06975 (5)	0.0231 (4)	
O6	0.89406 (19)	0.44735 (15)	0.20546 (5)	0.0208 (3)	
O7	0.6602 (2)	0.24246 (16)	0.03686 (5)	0.0278 (4)	
O8	0.7975 (2)	0.12148 (15)	0.07864 (5)	0.0217 (3)	
O9	1.3317 (2)	0.0814 (2)	0.10248 (7)	0.0412 (5)	
O10	0.4921 (2)	0.66853 (14)	0.23485 (5)	0.0219 (4)	

### Atomic displacement parameters (Å^2^)

**Table:**

	*U*^11^	*U*^22^	*U*^33^	*U*^12^	*U*^13^	*U*^23^
C1	0.0233 (11)	0.0216 (11)	0.0137 (9)	0.0018 (9)	−0.0003 (9)	−0.0023 (8)
C2	0.0207 (11)	0.0168 (10)	0.0172 (10)	−0.0010 (9)	−0.0009 (9)	−0.0035 (8)
C3	0.0237 (11)	0.0167 (10)	0.0163 (10)	−0.0004 (9)	0.0006 (9)	−0.0030 (8)
C4	0.0189 (11)	0.0180 (10)	0.0167 (10)	0.0014 (9)	0.0005 (9)	−0.0022 (8)
C5	0.0167 (10)	0.0174 (10)	0.0150 (10)	0.0016 (9)	0.0001 (8)	−0.0001 (8)
C6	0.0177 (11)	0.0216 (11)	0.0179 (10)	0.0006 (9)	0.0000 (9)	−0.0009 (9)
C7	0.0219 (11)	0.0136 (10)	0.0246 (11)	−0.0029 (9)	−0.0029 (9)	0.0004 (9)
C8	0.0186 (11)	0.0183 (10)	0.0152 (9)	0.0020 (9)	−0.0009 (9)	0.0005 (8)
C9	0.0182 (10)	0.0194 (10)	0.0140 (9)	0.0011 (9)	0.0016 (9)	0.0012 (8)
C10	0.0169 (10)	0.0191 (10)	0.0147 (10)	0.0015 (9)	0.0009 (8)	−0.0006 (8)
C11	0.0192 (10)	0.0170 (10)	0.0189 (10)	−0.0008 (9)	0.0026 (8)	0.0002 (8)
C12	0.0242 (12)	0.0157 (10)	0.0236 (11)	0.0002 (9)	0.0071 (9)	0.0024 (9)
C13	0.0218 (11)	0.0172 (10)	0.0172 (10)	0.0033 (9)	0.0030 (9)	0.0024 (8)
C14	0.0177 (10)	0.0163 (10)	0.0171 (10)	0.0018 (9)	0.0013 (8)	0.0008 (8)
C15	0.0216 (11)	0.0183 (10)	0.0162 (10)	0.0027 (9)	0.0031 (9)	0.0006 (8)
C16	0.0183 (11)	0.0245 (11)	0.0161 (10)	0.0004 (9)	0.0051 (9)	0.0001 (9)
C17	0.0241 (11)	0.0160 (10)	0.0193 (10)	0.0018 (9)	0.0013 (9)	0.0004 (8)
C18	0.0308 (13)	0.0244 (12)	0.0204 (11)	0.0066 (10)	0.0034 (10)	0.0038 (9)
C19	0.0219 (11)	0.0222 (11)	0.0160 (10)	0.0025 (10)	0.0052 (9)	0.0004 (8)
C20	0.0255 (12)	0.0209 (11)	0.0224 (11)	0.0031 (10)	0.0015 (9)	−0.0073 (9)
C21	0.0335 (15)	0.0531 (18)	0.0297 (13)	0.0206 (14)	0.0047 (12)	0.0016 (13)
C22	0.0263 (13)	0.0221 (12)	0.0388 (14)	0.0007 (10)	0.0089 (11)	0.0014 (10)
C23	0.0289 (14)	0.0248 (13)	0.0518 (17)	−0.0064 (12)	0.0125 (12)	−0.0126 (12)
C28	0.0245 (12)	0.0199 (11)	0.0237 (11)	0.0046 (10)	−0.0017 (9)	0.0000 (9)
C29	0.0251 (12)	0.0200 (11)	0.0198 (11)	0.0053 (10)	0.0014 (10)	−0.0025 (9)
C30	0.0179 (10)	0.0200 (10)	0.0178 (10)	0.0005 (9)	−0.0009 (9)	0.0001 (8)
C31	0.0377 (14)	0.0392 (14)	0.0160 (11)	−0.0076 (13)	−0.0023 (10)	−0.0010 (10)
C32	0.0224 (11)	0.0158 (10)	0.0220 (11)	0.0014 (9)	−0.0011 (9)	0.0047 (9)
C33	0.0217 (11)	0.0227 (11)	0.0206 (11)	0.0031 (10)	0.0000 (9)	0.0068 (9)
C34	0.0254 (12)	0.0260 (12)	0.0203 (10)	0.0061 (10)	−0.0013 (10)	0.0034 (9)
C35	0.0393 (15)	0.0390 (15)	0.0220 (12)	0.0101 (13)	−0.0014 (11)	−0.0037 (11)
C36	0.0282 (13)	0.0383 (14)	0.0260 (12)	−0.0044 (12)	0.0022 (11)	0.0097 (11)
O1	0.0276 (9)	0.0288 (9)	0.0153 (7)	0.0019 (8)	−0.0035 (7)	−0.0013 (7)
O2	0.0229 (8)	0.0194 (8)	0.0165 (7)	−0.0025 (7)	0.0030 (6)	−0.0014 (6)
O3	0.0325 (10)	0.0221 (8)	0.0251 (8)	−0.0089 (8)	−0.0013 (8)	−0.0023 (7)
O4	0.0222 (9)	0.0289 (9)	0.0306 (9)	−0.0016 (7)	−0.0080 (7)	0.0044 (7)
O5	0.0246 (8)	0.0280 (8)	0.0169 (7)	−0.0024 (8)	0.0000 (7)	−0.0037 (7)
O6	0.0211 (8)	0.0215 (8)	0.0199 (7)	0.0001 (7)	−0.0042 (7)	0.0008 (6)
O7	0.0291 (9)	0.0346 (9)	0.0197 (8)	0.0046 (8)	−0.0028 (7)	−0.0029 (7)
O8	0.0234 (8)	0.0201 (8)	0.0216 (8)	0.0005 (7)	0.0008 (7)	−0.0035 (6)
O9	0.0268 (10)	0.0582 (14)	0.0385 (11)	0.0118 (10)	−0.0020 (9)	−0.0137 (10)
O10	0.0281 (9)	0.0216 (8)	0.0160 (7)	0.0040 (7)	0.0015 (7)	−0.0048 (6)

### Geometric parameters (Å, °)

**Table:**

C1—O1	1.404 (3)	C16—O8	1.336 (3)
C1—O10	1.424 (3)	C17—O8	1.460 (3)
C1—C2	1.537 (3)	C17—C20	1.498 (3)
C1—C10	1.546 (3)	C17—H17	1.0000
C2—C30	1.514 (3)	C18—H18A	0.9800
C2—C3	1.545 (3)	C18—H18B	0.9800
C2—H2	1.0000	C18—H18C	0.9800
C3—O2	1.454 (3)	C19—H19A	0.9800
C3—C4	1.529 (3)	C19—H19B	0.9800
C3—H3	1.0000	C19—H19C	0.9800
C4—C29	1.525 (3)	C20—C21	1.350 (4)
C4—C28	1.527 (3)	C20—C22	1.429 (4)
C4—C5	1.559 (3)	C21—O9	1.351 (4)
C5—C6	1.530 (3)	C21—H21	0.9500
C5—C10	1.576 (3)	C22—C23	1.350 (4)
C5—H5	1.0000	C22—H22	0.9500
C6—C7	1.510 (3)	C23—O9	1.362 (4)
C6—H6A	0.9900	C23—H23	0.9500
C6—H6B	0.9900	C28—H28A	0.9800
C7—O4	1.202 (3)	C28—H28B	0.9800
C7—O5	1.339 (3)	C28—H28C	0.9800
C8—O6	1.472 (3)	C29—O10	1.429 (3)
C8—C30	1.472 (3)	C29—H29A	0.9900
C8—C14	1.518 (3)	C29—H29B	0.9900
C8—C9	1.527 (3)	C30—O6	1.455 (3)
C9—C11	1.542 (3)	C30—H30	1.0000
C9—C10	1.557 (3)	C31—O5	1.455 (3)
C9—H9	1.0000	C31—H31A	0.9800
C10—C19	1.539 (3)	C31—H31B	0.9800
C11—C12	1.525 (3)	C31—H31C	0.9800
C11—H11A	0.9900	C32—O3	1.209 (3)
C11—H11B	0.9900	C32—O2	1.349 (3)
C12—C13	1.561 (3)	C32—C33	1.486 (3)
C12—H12A	0.9900	C33—C34	1.339 (3)
C12—H12B	0.9900	C33—C36	1.503 (3)
C13—C14	1.536 (3)	C34—C35	1.488 (3)
C13—C18	1.540 (3)	C34—H34	0.9500
C13—C17	1.541 (3)	C35—H35A	0.9800
C14—C15	1.549 (3)	C35—H35B	0.9800
C14—H14	1.0000	C35—H35C	0.9800
C15—C16	1.511 (3)	C36—H36A	0.9800
C15—H15A	0.9900	C36—H36B	0.9800
C15—H15B	0.9900	C36—H36C	0.9800
C16—O7	1.213 (3)	O1—H1	0.8400
			
O1—C1—O10	102.93 (16)	H15A—C15—H15B	107.2
O1—C1—C2	111.83 (19)	O7—C16—O8	118.3 (2)
O10—C1—C2	108.37 (18)	O7—C16—C15	121.4 (2)
O1—C1—C10	112.88 (18)	O8—C16—C15	120.26 (19)
O10—C1—C10	110.26 (18)	O8—C17—C20	109.06 (18)
C2—C1—C10	110.26 (17)	O8—C17—C13	110.62 (18)
C30—C2—C1	110.99 (18)	C20—C17—C13	116.84 (19)
C30—C2—C3	113.69 (18)	O8—C17—H17	106.6
C1—C2—C3	107.30 (18)	C20—C17—H17	106.6
C30—C2—H2	108.2	C13—C17—H17	106.6
C1—C2—H2	108.2	C13—C18—H18A	109.5
C3—C2—H2	108.2	C13—C18—H18B	109.5
O2—C3—C4	106.98 (17)	H18A—C18—H18B	109.5
O2—C3—C2	112.33 (18)	C13—C18—H18C	109.5
C4—C3—C2	109.95 (18)	H18A—C18—H18C	109.5
O2—C3—H3	109.2	H18B—C18—H18C	109.5
C4—C3—H3	109.2	C10—C19—H19A	109.5
C2—C3—H3	109.2	C10—C19—H19B	109.5
C29—C4—C28	109.30 (18)	H19A—C19—H19B	109.5
C29—C4—C3	104.77 (18)	C10—C19—H19C	109.5
C28—C4—C3	110.43 (19)	H19A—C19—H19C	109.5
C29—C4—C5	109.33 (18)	H19B—C19—H19C	109.5
C28—C4—C5	115.08 (18)	C21—C20—C22	105.0 (2)
C3—C4—C5	107.43 (18)	C21—C20—C17	126.1 (2)
C6—C5—C4	113.65 (19)	C22—C20—C17	129.0 (2)
C6—C5—C10	113.09 (18)	C20—C21—O9	111.8 (3)
C4—C5—C10	109.14 (18)	C20—C21—H21	124.1
C6—C5—H5	106.8	O9—C21—H21	124.1
C4—C5—H5	106.8	C23—C22—C20	106.9 (3)
C10—C5—H5	106.8	C23—C22—H22	126.6
C7—C6—C5	115.98 (19)	C20—C22—H22	126.6
C7—C6—H6A	108.3	C22—C23—O9	110.2 (3)
C5—C6—H6A	108.3	C22—C23—H23	124.9
C7—C6—H6B	108.3	O9—C23—H23	124.9
C5—C6—H6B	108.3	C4—C28—H28A	109.5
H6A—C6—H6B	107.4	C4—C28—H28B	109.5
O4—C7—O5	124.0 (2)	H28A—C28—H28B	109.5
O4—C7—C6	124.0 (2)	C4—C28—H28C	109.5
O5—C7—C6	111.90 (19)	H28A—C28—H28C	109.5
O6—C8—C30	59.23 (13)	H28B—C28—H28C	109.5
O6—C8—C14	112.87 (18)	O10—C29—C4	111.23 (18)
C30—C8—C14	118.07 (19)	O10—C29—H29A	109.4
O6—C8—C9	113.87 (17)	C4—C29—H29A	109.4
C30—C8—C9	120.70 (19)	O10—C29—H29B	109.4
C14—C8—C9	117.45 (18)	C4—C29—H29B	109.4
C8—C9—C11	109.77 (17)	H29A—C29—H29B	108.0
C8—C9—C10	113.02 (18)	O6—C30—C8	60.37 (14)
C11—C9—C10	114.46 (18)	O6—C30—C2	113.99 (18)
C8—C9—H9	106.3	C8—C30—C2	121.8 (2)
C11—C9—H9	106.3	O6—C30—H30	116.2
C10—C9—H9	106.3	C8—C30—H30	116.2
C19—C10—C1	108.32 (17)	C2—C30—H30	116.2
C19—C10—C9	109.48 (18)	O5—C31—H31A	109.5
C1—C10—C9	108.61 (17)	O5—C31—H31B	109.5
C19—C10—C5	114.11 (18)	H31A—C31—H31B	109.5
C1—C10—C5	106.07 (17)	O5—C31—H31C	109.5
C9—C10—C5	110.08 (17)	H31A—C31—H31C	109.5
C12—C11—C9	111.02 (19)	H31B—C31—H31C	109.5
C12—C11—H11A	109.4	O3—C32—O2	122.4 (2)
C9—C11—H11A	109.4	O3—C32—C33	124.2 (2)
C12—C11—H11B	109.4	O2—C32—C33	113.45 (19)
C9—C11—H11B	109.4	C34—C33—C32	120.5 (2)
H11A—C11—H11B	108.0	C34—C33—C36	124.6 (2)
C11—C12—C13	114.63 (18)	C32—C33—C36	114.5 (2)
C11—C12—H12A	108.6	C33—C34—C35	125.3 (2)
C13—C12—H12A	108.6	C33—C34—H34	117.3
C11—C12—H12B	108.6	C35—C34—H34	117.3
C13—C12—H12B	108.6	C34—C35—H35A	109.5
H12A—C12—H12B	107.6	C34—C35—H35B	109.5
C14—C13—C18	109.84 (19)	H35A—C35—H35B	109.5
C14—C13—C17	108.53 (17)	C34—C35—H35C	109.5
C18—C13—C17	109.13 (18)	H35A—C35—H35C	109.5
C14—C13—C12	111.65 (18)	H35B—C35—H35C	109.5
C18—C13—C12	109.20 (18)	C33—C36—H36A	109.5
C17—C13—C12	108.44 (19)	C33—C36—H36B	109.5
C8—C14—C13	111.67 (17)	H36A—C36—H36B	109.5
C8—C14—C15	111.12 (18)	C33—C36—H36C	109.5
C13—C14—C15	112.00 (18)	H36A—C36—H36C	109.5
C8—C14—H14	107.2	H36B—C36—H36C	109.5
C13—C14—H14	107.2	C1—O1—H1	109.5
C15—C14—H14	107.2	C32—O2—C3	115.97 (17)
C16—C15—C14	117.57 (18)	C7—O5—C31	115.3 (2)
C16—C15—H15A	107.9	C30—O6—C8	60.40 (13)
C14—C15—H15A	107.9	C16—O8—C17	120.65 (17)
C16—C15—H15B	107.9	C21—O9—C23	106.2 (2)
C14—C15—H15B	107.9	C1—O10—C29	112.70 (15)
			
O1—C1—C2—C30	73.3 (2)	C9—C8—C14—C15	85.2 (2)
O10—C1—C2—C30	−173.89 (17)	C18—C13—C14—C8	−69.2 (2)
C10—C1—C2—C30	−53.1 (2)	C17—C13—C14—C8	171.59 (18)
O1—C1—C2—C3	−161.90 (17)	C12—C13—C14—C8	52.1 (2)
O10—C1—C2—C3	−49.1 (2)	C18—C13—C14—C15	165.47 (18)
C10—C1—C2—C3	71.6 (2)	C17—C13—C14—C15	46.2 (2)
C30—C2—C3—O2	−12.4 (3)	C12—C13—C14—C15	−73.2 (2)
C1—C2—C3—O2	−135.55 (18)	C8—C14—C15—C16	−133.7 (2)
C30—C2—C3—C4	106.6 (2)	C13—C14—C15—C16	−8.0 (3)
C1—C2—C3—C4	−16.5 (2)	C14—C15—C16—O7	162.6 (2)
O2—C3—C4—C29	−171.39 (17)	C14—C15—C16—O8	−18.8 (3)
C2—C3—C4—C29	66.4 (2)	C14—C13—C17—O8	−62.9 (2)
O2—C3—C4—C28	−53.8 (2)	C18—C13—C17—O8	177.45 (18)
C2—C3—C4—C28	−176.06 (17)	C12—C13—C17—O8	58.6 (2)
O2—C3—C4—C5	72.4 (2)	C14—C13—C17—C20	62.7 (3)
C2—C3—C4—C5	−49.8 (2)	C18—C13—C17—C20	−57.0 (3)
C29—C4—C5—C6	85.0 (2)	C12—C13—C17—C20	−175.86 (19)
C28—C4—C5—C6	−38.5 (3)	O8—C17—C20—C21	−145.8 (2)
C3—C4—C5—C6	−161.87 (17)	C13—C17—C20—C21	87.9 (3)
C29—C4—C5—C10	−42.3 (2)	O8—C17—C20—C22	32.4 (3)
C28—C4—C5—C10	−165.69 (18)	C13—C17—C20—C22	−93.9 (3)
C3—C4—C5—C10	70.9 (2)	C22—C20—C21—O9	0.6 (3)
C4—C5—C6—C7	76.8 (2)	C17—C20—C21—O9	179.2 (2)
C10—C5—C6—C7	−158.06 (19)	C21—C20—C22—C23	−0.4 (3)
C5—C6—C7—O4	−146.4 (2)	C17—C20—C22—C23	−178.9 (2)
C5—C6—C7—O5	36.0 (3)	C20—C22—C23—O9	0.1 (3)
O6—C8—C9—C11	−149.39 (18)	C28—C4—C29—O10	−168.90 (19)
C30—C8—C9—C11	143.4 (2)	C3—C4—C29—O10	−50.6 (2)
C14—C8—C9—C11	−14.3 (3)	C5—C4—C29—O10	64.3 (2)
O6—C8—C9—C10	81.5 (2)	C14—C8—C30—O6	−101.3 (2)
C30—C8—C9—C10	14.3 (3)	C9—C8—C30—O6	101.2 (2)
C14—C8—C9—C10	−143.36 (19)	O6—C8—C30—C2	−101.4 (2)
O1—C1—C10—C19	61.0 (2)	C14—C8—C30—C2	157.3 (2)
O10—C1—C10—C19	−53.5 (2)	C9—C8—C30—C2	−0.2 (3)
C2—C1—C10—C19	−173.09 (18)	C1—C2—C30—O6	−49.4 (2)
O1—C1—C10—C9	−57.8 (2)	C3—C2—C30—O6	−170.44 (17)
O10—C1—C10—C9	−172.29 (16)	C1—C2—C30—C8	19.5 (3)
C2—C1—C10—C9	68.1 (2)	C3—C2—C30—C8	−101.6 (2)
O1—C1—C10—C5	−176.07 (18)	O3—C32—C33—C34	168.5 (2)
O10—C1—C10—C5	69.4 (2)	O2—C32—C33—C34	−10.0 (3)
C2—C1—C10—C5	−50.2 (2)	O3—C32—C33—C36	−5.3 (3)
C8—C9—C10—C19	−164.78 (18)	O2—C32—C33—C36	176.15 (19)
C11—C9—C10—C19	68.6 (2)	C32—C33—C34—C35	−170.6 (2)
C8—C9—C10—C1	−46.7 (2)	C36—C33—C34—C35	2.6 (4)
C11—C9—C10—C1	−173.34 (17)	O3—C32—O2—C3	3.1 (3)
C8—C9—C10—C5	69.0 (2)	C33—C32—O2—C3	−178.40 (18)
C11—C9—C10—C5	−57.6 (2)	C4—C3—O2—C32	159.42 (17)
C6—C5—C10—C19	−26.6 (3)	C2—C3—O2—C32	−79.9 (2)
C4—C5—C10—C19	101.0 (2)	O4—C7—O5—C31	−0.4 (3)
C6—C5—C10—C1	−145.73 (18)	C6—C7—O5—C31	177.3 (2)
C4—C5—C10—C1	−18.2 (2)	C2—C30—O6—C8	114.2 (2)
C6—C5—C10—C9	97.0 (2)	C14—C8—O6—C30	110.1 (2)
C4—C5—C10—C9	−135.48 (19)	C9—C8—O6—C30	−112.7 (2)
C8—C9—C11—C12	58.2 (2)	O7—C16—O8—C17	−178.86 (19)
C10—C9—C11—C12	−173.53 (17)	C15—C16—O8—C17	2.5 (3)
C9—C11—C12—C13	−46.5 (3)	C20—C17—O8—C16	−91.0 (2)
C11—C12—C13—C14	−8.9 (3)	C13—C17—O8—C16	38.8 (3)
C11—C12—C13—C18	112.8 (2)	C20—C21—O9—C23	−0.6 (3)
C11—C12—C13—C17	−128.4 (2)	C22—C23—O9—C21	0.3 (3)
O6—C8—C14—C13	94.9 (2)	O1—C1—O10—C29	−172.35 (18)
C30—C8—C14—C13	161.04 (19)	C2—C1—O10—C29	69.1 (2)
C9—C8—C14—C13	−40.7 (3)	C10—C1—O10—C29	−51.7 (2)
O6—C8—C14—C15	−139.27 (18)	C4—C29—O10—C1	−15.1 (3)
C30—C8—C14—C15	−73.1 (3)		

### Hydrogen-bond geometry (Å, °)

**Table:**

*D*—H···*A*	*D*—H	H···*A*	*D*···*A*	*D*—H···*A*
O1—H1···O6	0.84	2.08	2.774 (2)	139
